# Pulmonary Histoplasmosis Mimicking Lung Cancer

**DOI:** 10.5334/jbsr.3563

**Published:** 2024-04-22

**Authors:** Ramazan Orkun Onder, Emel Agca Nasuhbeyoglu, Tümay Bekci

**Affiliations:** 1 Faculty of Medicine, Department of Radiology, Giresun University, Giresun, Turkey; 2 Faculty of Medicine, Department of Radiology, Giresun University, Giresun, Turkey; 3 Faculty of Medicine, Department of Radiology, Giresun University, Giresun, Turkey

**Keywords:** Histoplasma capsulatum, pulmonary, histoplasmosis, lung cancer

## Abstract

*Teaching point:* Due to the mass-like appearance of pulmonary histoplasmosis in the lung, radiological misdiagnosis may occur. Fungal infections should be considered in the differential diagnosis, especially in immune-compromised patients.

## Case History

**Figure 1:**
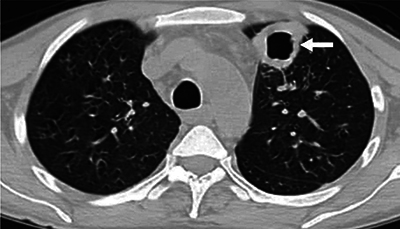
Non-contrast CT axial sections show a subpleural thick-walled cavitary lesion in the anterior segment of the upper lobe of the left lung (white arrow).

**Figure 2:**
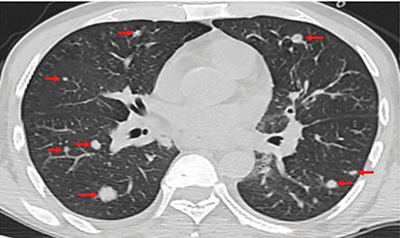
The lung parenchyma shows multiple consolidated nodular areas on non-contrast CT axial slices (red arrows).

A 43-year-old man was admitted to the infectious diseases clinic with complaints of cough, shortness of breath, and weight loss. He was an inmate with a history of acquired immunodeficiency, was not taking antiretrovirals, and had a CD4 count of 147 μL. Chest computed tomography (CT) scans revealed a thick-walled cavitary lesion in the anterior segment of the upper lobe of the left lung (Figure 1) and multiple areas of nodular consolidation in both lungs (Figure 2). Initially, the cavitary lesion was suspected to be a primary lung tumor, while the multiple nodular areas were considered as metastases. However, histopathological and fluid-based cytological examinations of the cavitary lesion in the interventional radiology clinic revealed areas rich in epithelioid histiocytes with foci of necrosis and numerous red, round-shaped microorganisms staining positively with Gomori methenamine silver (GMS) and Periodic acid–Schiff (PAS), suggesting *Histoplasma capsulatum* infection.

## Comments

*H. capsulatum*, the causative agent of histoplasmosis, is a dimorphic fungus found in soils worldwide, predominantly in central and eastern North America. More than half of adults living in endemic areas are infected with *H. Capsulatum* [1]. Although most cases remain subclinical, histoplasmosis can have a spectrum of clinical manifestations ranging from flu-like symptoms to acute respiratory distress syndrome, shock, and death. In cases of acute pulmonary histoplasmosis, imaging typically shows solitary or multiple nodules. Chronic cavitary pulmonary histoplasmosis may develop after acute pulmonary histoplasmosis and is characterized by persistent cavitation, fibrosis, and progressive lung failure [2]. Nodules in acute histoplasmosis have a nonspecific appearance and may sometimes show cavitation or a ground-glass halo. They may therefore mimic metastases [3].
